# Parental divorce and nicotine addiction in Lebanese adolescents: the mediating role of child abuse and bullying victimization

**DOI:** 10.1186/s13690-022-00848-9

**Published:** 2022-03-14

**Authors:** Elie Bou Sanayeh, Katia Iskandar, Marie-Claude Fadous Khalife, Sahar Obeid, Souheil Hallit

**Affiliations:** 1 School of Medicine and Medical Sciences, Holy Spirit University of Kaslik, P.O. Box 446, Jounieh, Lebanon; 2 Department of Mathématiques Informatique et Télécommunications, Université Toulouse III, Paul Sabatier, INSERM, UMR 1027, F-31000 Toulouse, France; 3 Department of Pediatrics, Notre Dame des Secours University Hospital Center, Street 93, Byblos, Postal Code 3 Lebanon; 4 School of Arts and Sciences, Social and Education Sciences Department, Lebanese American University, Jbeil, Lebanon; 5 Research Department, Psychiatric Hospital of the Cross, Jal Eddib, Lebanon

**Keywords:** Adolescents, Bullying victimization, Child abuse, Cigarette dependence, Lebanon, Nicotine addiction, Parental divorce, Smoking, Waterpipe dependence

## Abstract

**Background:**

Lebanon ranks first amongst Middle-Eastern countries in terms of cigarette and waterpipe smoking. Understanding the mediating factors for nicotine addiction in adolescents who have experienced parental divorce is vital to take effective measures that will help in lowering its prevalence in our community. The objective of this study was to investigate the association between the increasingly concerning parental divorce and nicotine addiction in Lebanese adolescents while taking into consideration the plausible mediating effect of abuse and bullying victimization.

**Methods:**

This was a cross-sectional survey-based study that was conducted between January and May 2019. A total of 1810 adolescents aged between 14 and 17 years was enrolled from 16 Lebanese schools. Linear regressions taking the cigarette and waterpipe dependence scores as dependent variables were conducted respectively, using the SPSS software. PROCESS v3.4 model 4 was used for mediation analysis.

**Results:**

A total of 11.9% of the enrolled participants had divorced parents. Higher cigarette and waterpipe dependence were found in adolescents whose parents were divorced compared to those living together. More child psychological abuse, having divorced parents vs living together, and more child physical abuse were significantly associated with higher cigarette dependence. More child psychological and physical, and having divorced parents vs living together were significantly associated with more waterpipe dependence. In addition, all forms of abuse (except neglect) and bullying victimization had a partially mediating effect in the associations between parental divorce and nicotine dependence (cigarette and waterpipe) in adolescents.

**Conclusion:**

This study results may serve as a first step towards enrolling separated parents and their children in special prevention programs to help them create a protective and supportive environment.

## Background

Following the Lebanese financial crisis, the dramatic collapse in basic services and the increase in poverty and unemployment rates, Lebanon has faced a 101% increase in parental divorce rates between 2006 and 2017 [1]. The country is now considered to have the fourth highest level of divorces in the Arab world [2], making it of major interest to study the influence of parental separation on their vulnerable offspring [3]. Based on the Problem Behavior Theory and Bowlby’s early attachment theory, several studies have shown that adolescents who experience insecure attachment secondary to the separation from a parent, might exhibit social, psychological, or behavioral disruption [3–6]. This increase in risk behaviors, includes but is not limited, to cigarette and waterpipe addiction [3, 4, 7]. In fact, Lebanese, European and American studies have reported higher rates of nicotine addiction in adolescents who have experienced parental divorce at an early age, when compared to those who lived with both parents [4, 6, 7].

Nevertheless, vulnerability varies among adolescents, and not every exposed child is at equal risk of developing addiction [8] given that other factors may play a mediating role [9]. Many studies have demonstrated that an accumulation of multiple adverse childhood experiences may contribute to nicotine addiction later on [10, 11]. For instance, the deleterious and widespread negative impact of child neglect, abuse (physical, sexual, and psychological), bullying victimization, low physical activity or household crowding, on mental health outcomes and risk behaviors, have become increasingly recognized in large part [9–12].

Both abuse and bullying victimization were lately being considered as nationally important social challenges, given their elevated prevalence with respectively 48 [13] and 50% [14] in our society. However, it is difficult to determine the real prevalence of abuse in Lebanon, given the Lebanese culture of heavy-handed parenting with corporal punishment, and lack of legal systems that command disclosing child maltreatment [13]. Same applies to bullying with the absence of anti-bullying rules, awareness programs, and bullying-prevention strategies in our Lebanese schools [14]. Previous studies have also showed that these two entities were more prevalent among children living in chaotic family environments [13, 14]. It is plausible that the lengthy lack of parental companionship, care, and guarding with its subsequent emotional sensitivity, difficulty adapting to change, and loss of interest in social activities makes children of divorced parents more vulnerable to various forms of abuses and bullying behavior [3–6, 13, 14]. With the accumulation of these negative experiences comes nicotine dependence as a coping mechanism, given its strong psychological, cultural, and social symbolism as a marker of resilience, autonomy, and precocity [6].

Yet, to our knowledge, no local studies have evaluated the influence of abuse and bullying victimization as mediating factors on nicotine addiction among Lebanese adolescents who have experienced parental divorce despite the fact that dependence rates are steadily growing in Lebanon [15]. As reported by the “United Nations Development Programme” (UNDP) in 2015, Lebanon has the highest rates of cigarette consumption in the Middle East, with 27.7 to 50% of his adults being cigarette smokers [15–17]. This has been exacerbated by the fact that Lebanese tax rates on tobacco are suboptimal making it the cheapest place in the Arab region to buy cigarettes, as well as the lack of laws that control smoking in public places [15]. In addition, in 2016, Lebanon was ranked first among 25 Eastern European and Eastern Mediterranean countries in waterpipe usage, with 36.9% of his teens having used it [4, 16, 18–20]. Factors contributing to its popularity include its aromatic smell, its popularity in the Arabic culture, the uncontrolled marketing and promotion campaigns, and most importantly the lack of knowledge [21–23] and common Lebanese belief that waterpipe smoking has minimal adverse effects on health given that smoke is being filtered by the water [19, 24].

It is expected that such an increase in smoking rates among adolescents will not only have serious repercussions for the medical system in Lebanon given the consequential life-threatening medical events, but also it will profoundly affect the social and juridical aspects of the community given that smoking anticipates the transition of adolescents into various delinquent behaviors, by outing with substance-using peers and manifesting what is called the negative identity, that lowers their self-control and makes them unable to refrain from danger situations [4, 6, 7, 13, 21].

**Fig. 1:**
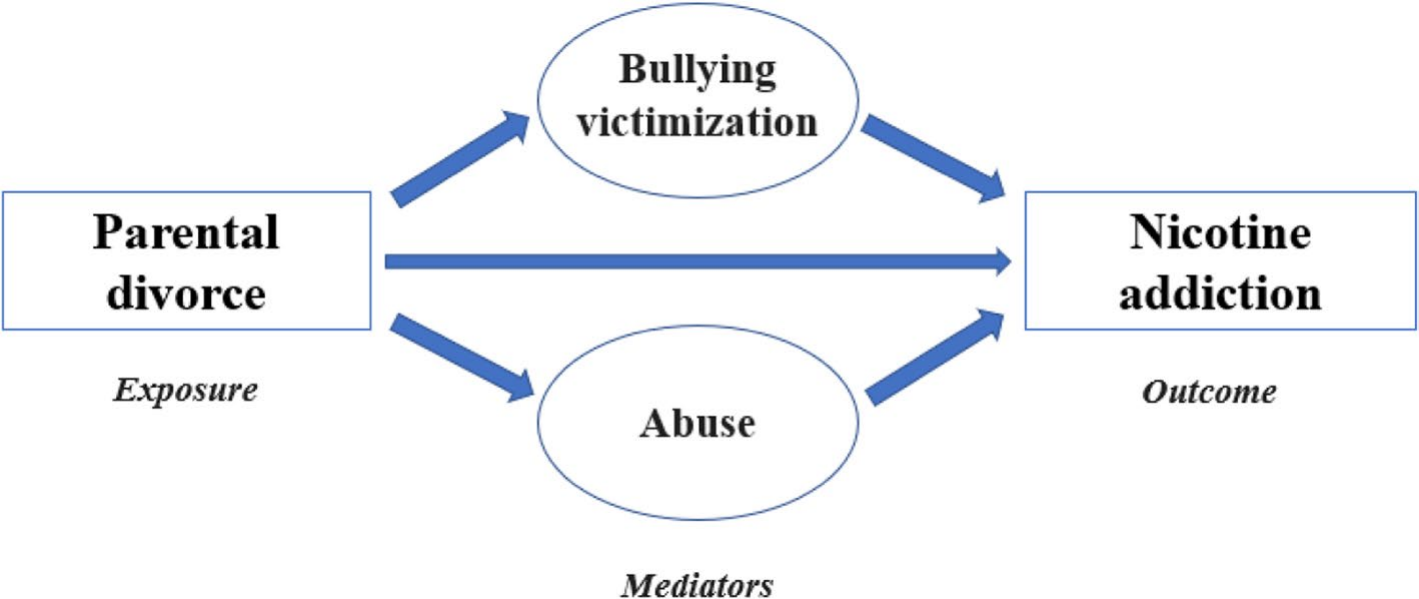
A visualization showing the pathways from the exposure (parental divorce) to the mediators (bullying victimization and abuse), then to the outcome (nicotine addiction)

Therefore, understanding the mediating factors for nicotine addiction in adolescents who have experienced parental divorce is vital to take effective measures that will help in avoiding these triggers and ultimately lowering smoking prevalence in our community. In the current study we aimed to evaluate for the first time in the Lebanese community, the association between parental divorce and nicotine dependence among Lebanese adolescents, with a mediating role for child abuse and bullying victimization (Fig. 1). We hypothesize that parental divorce would be associated with more nicotine dependence and that this association would be mediated by at least one form of child abuse.

## Methods

### Participants

This was a survey-based cross-sectional study that was conducted between January and May 2019. Eligible participants were adolescents aged between 14 and 17 years, selected from schools allocated in all eight Lebanese governorates (Muhafazah). Based on a list provided by “The Ministry of Education and Higher Education” of all currently operating Lebanese schools, and based on each governorate’s population density, we adopted a proportionate random sampling to enroll the schools. Out of the 18 approached institutions; 16 have welcomed the idea and accepted to participate. The distribution was as follow: six schools from Mount Lebanon, two from Beirut, two from Beqaa, two from North Lebanon and two from South Lebanon. Excluded were those who refused to participate in this study. The methodology adopted in this study is close to the one used in previous papers [4, 14, 25–30].

### Minimal sample size calculation

Taking into consideration that twenty factors were included in the multivariable analysis and based on a power of 80%, an alpha error of 5% and an effect size f2 = 2%, the G-power software have calculated a minimal sample of 395 subjects.

### Questionnaire

The main study tool was a self-administered questionnaire written in Arabic, Lebanon’s native language and it required approximately 50 to 60 min to be filled. In order to eliminate any parental involvement or influencing factor, the questionnaire had to be filled at school during a free classroom session. All responses were confidential and anonymous.

The questionnaire was divided into two sections: The first one evaluated participants’ sociodemographic characteristics including whether their parents are currently divorced or not; as well as other general considerations that allowed us to calculate the Body Mass Index (BMI) (calculated based on self-reported heights and weights of participants - kg/m^2^), the household crowding index (HCI) (number of persons constantly living in that house divided by the number of rooms in the participant’s house after excluding the kitchen and bathrooms [31] (higher HCI indicates lower socioeconomic status), and the total Physical Activity Index (by multiplying the self-reported intensity, duration, and frequency of daily activity) [32]. The second section evaluated participants’ waterpipe and nicotine dependence, as well as lifetime exposure to bullying victimization and child abuse, using respectively the following scales:

- *Lebanon Waterpipe Dependence Scale-11 (LWDS-11)*

The LWDS-11 scale [33, 34] is a Lebanese validated scale that was used to detect waterpipe dependence. It consisted of eleven items covering four subscales: nicotine dependence, negative reinforcement, psychological craving, and positive reinforcement (e.g., smoking for pleasure, smoking alone, number of waterpipes smoked per day, etc.). Each item was rated on a 4-point Likert-type scale with scores ranging from 0 to 3 for each question and an overall score ranging from 0 to 33. Subjects were considered as having dependence to waterpipe when scoring 10 or more [33]. In the conducted study, the Cronbach’s alpha was 0.888.

- *Fagerström Test for Nicotine Dependence (FTND)*

The FTND scale is a validated standard assessment tool that was used to evaluate the level of physical addiction to nicotine induced by cigarette smoking [35]. It consisted of six items that assessed the number of cigarettes consumed per day, the level of nicotine dependence and the compulsion to smoke. Four items were a yes/no questions thus rated from 0 to 1; and the two remaining were multiple-choice questions rated from 0 to 3 on a 4-point Likert-type scale. The overall score of the FTND is ten and only one positive answer was required to indicate the presence of physical dependence to nicotine but the higher the overall score, the more intense was the dependence. Subsequently, dependence ranged from low (when scoring 1 or 2) to high (when scoring ≥8) [35]. In the conducted study, Cronbach’s alpha was 0.825.

- *The Illinois Bully scale (IBS)*

The IBS scale, validated in Lebanon [14], was used to detect the frequency of bullying victimization and perpetration during the month that preceded the day of the evaluation. It consisted of eighteen items, each one rated on a 5-point Likert-type scale from 0 (never) to 4 (very often) and it covered three subscales (victimization, bullying and fighting). A higher score on each subscale denoted a greater tendency of engaging in the accordingly evaluated behavior [14]. In the conducted study, Cronbach’s alpha was 0.975.

- *Child Abuse Self-Report Scale (CASRS)*

The CASRS is a validated tool [36, 37] that was used to reveal the presence of lifetime child abuse. It consisted of 38 items, each one rated on a 4-point Likert-type scale from 0 (never) to 3 (always), covering the four types of abuse: 8 questions investigated the physical abuse, 14 the emotional/psychological abuse, 5 the sexual abuse and 11 the neglect. Higher scores were indicative of a higher degree of child abuse in all subscales [36, 37]. In the conducted study, Cronbach’s alphas were respectively for each type of the abovementioned abuses 0.966, 0.973, 0.954 and 0.971.

### Forward and backward translation

For all non-validated scales, forward and backward translation were performed by two translators. The first one from English into Arabic and the second one back into the original language. At last, inconsistencies and discrepancies found during comparison of the back-translated English version to the original English form, were solved by consensus.

### Statistical analysis

The analysis of all data was done using SPSS software version 25. The internal consistency of all used scales was evaluated and the Cronbach’s alpha values were recorded. Missing values constituted less than 5% of the total data and thus were not replaced. Student t test was used to compare two means, whereas Pearson test was used to correlate two continuous variables. According to Cohen, d = 0.2 was considered as a small effect size, 0.5 represented a ‘medium’ effect size and 0.8 a ‘large’ effect size [38]. Two linear regressions were conducted, taking the cigarette and waterpipe dependence scores as dependent variables respectively.

PROCESS v3.4 model 4 was used for mediation analysis. Pathway A determined the regression coefficient for the effect of parental divorce on the mediators (child physical/sexual/psychological abuse, child neglect, bullying victimization), Pathway B examined the association between the mediators on cigarette/waterpipe dependence, independent of the psychological distress, and Pathway C′ estimated the total and direct effect of parental divorce on cigarette/waterpipe dependence. Pathway AB calculated the indirect intervention effects. Significance was assumed when the confidence interval did not include zero [39]. Independent variables entered in the linear regressions and in the mediation analysis models were those that showed an effect size or a correlation coefficient ≥ │0.24│to achieve more parsimonious models [40]. Significance was set at a *p* < 0.05.

## Results

Out of two thousand distributed questionnaires, 1810 were collected back and sent for data entry, achieving a response rate of 90.5%. Table 1 summarizes the sociodemographic characteristics of the participants. The mean age was 15.41 ± 1.14 years, with 53.3% of participants being females. Additionally, 11.9% of adolescents had separated/divorced parents.

**Table 1:** Sociodemographic characteristics of the 1810 enrolled Lebanese adolescents aged between 14 and 17 years from 16 schools allocated in all 8 Lebanese governorates

	**Frequency (%)**
**Gender**	
Male	844 (46.7%)
Female	963 (53.3%)
**Parental status**	
Living together	1581 (88.1%)
Divorced	213 (11.9%)
	**Mean ± SD**
**Age (in years)**	15.41 ± 1.14
**Household crowding index**	1.00 ± 0.64
**Waterpipe dependence score**	4.73 ± 8.68
**Cigarette dependence score**	1.53 ± 2.83
**Child physical abuse**	5.74 ± 6.70
**Child sexual abuse**	2.79 ± 4.01
**Child psychological abuse**	10.64 ± 11.49
**Child neglect**	13.92 ± 10.71
**Bullying victimization**	6.59 ± 7.12

Note. Numbers might not add to the full sample size because of missing values

### Bivariate analysis

Higher cigarette and waterpipe dependence were found in adolescents whose parents were divorced compared to those whom parents were living together, and were significantly and positively associated with more child physical, sexual and psychological abuse, child neglect, and bullying victimization. Furthermore, it was negatively but weakly associated with older age and a higher household crowding index (Table 2).

**Table 2:** Bivariate analysis taking the cigarette and waterpipe dependence scores as the dependent variables

	**FTND**	***p*-value**	**LWDS**	***p*-value**
	**Mean ± SD**		**Mean ± SD**	
**Gender**				
Male	1.50 ± 2.74	0.648	5.00 ± 8.87	0.225
Female	1.56 ± 2.91		4.50 ± 8.51	
Effect size	0.021		0.057	
**Parental status**				
Living together	1.22 ± 2.52	**< 0.001**	4.09 ± 8.37	**< 0.001**
Divorced	3.90 ± 3.77		9.65 ± 9.47	
Effect size	0.835		0.622	
	**Correlation coefficient**	***p*-value**	**Correlation coefficient**	***p*-value**
Child physical abuse	0.387	**< 0.001**	0.259	**< 0.001**
Child sexual abuse	0.309	**< 0.001**	0.231	**< 0.001**
Child psychological abuse	0.464	**< 0.001**	0.285	**< 0.001**
Child neglect	0.185	**< 0.001**	0.228	**< 0.001**
Bullying victimization	0.253	**< 0.001**	0.205	**< 0.001**
Age	−0.147	**< 0.001**	−0.152	**< 0.001**
Household crowding index	−0.089	**< 0.001**	− 0.081	**0.001**
Physical activity score	−0.042	0.097	−0.021	0.411

Note. Numbers in bold indicate significant *p*-values; FTND: Fagerstrom Test for Nicotine Dependence, LWDS-11: Lebanon Waterpipe dependence scale – 11

### Multivariable analysis

The results of a first linear regression, taking the cigarette dependence scale (FTND scale) as the dependent variable, showed that more child psychological abuse (B = 0.09; *p* < 0.001; 95% CI 0.08–0.11), having divorced parents vs living together (B = 1.92; *p* < 0.001; 95% CI 1.55–2.29), and more child physical abuse (B = 0.03; *p* = 0.04; 95% CI 0.001–0.06) were significantly associated with higher cigarette dependence (higher FTND scores) (Table 3, Model 1).

**Table 3:** Multivariable analysis

**Model 1: Linear regression taking the FTND scale as the dependent variable.**					
**Variable**	**Unstandardized Beta**	**Standardized Beta**	***p***	**95% Confidence Interval**	
Child psychological abuse	0.09	0.38	**< 0.001**	0.08	0.11
Parental status (divorced vs living together*)	1.92	0.22	**< 0.001**	1.55	2.29
Child physical abuse	0.03	0.07	**0.04**	0.001	0.06
***Note. Variables entered in the models****: parental status, child physical abuse, child sexual abuse, child psychological abuse, bullying victimization.* *Adjusted R*^*2*^ *= 27.5%, p < 0.001.*					
**Model 2: Linear regression taking the LWDS-11 as the dependent variable.**					
**Variable**	**Unstandardized Beta**	**Standardized Beta**	***p***	**95% Confidence Interval**	
Child psychological abuse	0.15	0.19	**< 0.001**	0.09	0.20
Parental status (divorced vs living together*)	3.90	0.15	**< 0.001**	2.64	5.16
Child physical abuse	0.11	0.08	**0.020**	0.02	0.20
*Note. Numbers in bold indicate significant p-values;* ***Variables entered in the model:*** *parental status, child physical abuse, child psychological abuse.* *Adjusted R*^*2*^ *= 26.1%; p < 0.001.*					

The results of a second linear regression, taking the waterpipe dependence scale (LWDS-11 scale) as the dependent variable, showed that more child psychological (B = 0.15; *p* < 0.001; 95% CI 0.09–0.20) and physical (B = 0.11; *p* = 0.020; 95% CI 0.02–0.20), and having divorced parents vs living together (B = 3.90; *p* < 0.001; 95% CI 2.64–5.16) were significantly associated with more waterpipe dependence (higher LWDS-11 scores) (Table 3, Model 2).

### Mediation analysis

Child physical, sexual and psychological abuse, and bullying victimization partially mediated the association between parental divorce and cigarette dependence (Table 4, Model 1) as well as between parental divorce and waterpipe dependence (Table 4, Model 2).

**Table 4:** Mediation analysis

**Model 1: Cigarette dependence score as the dependent variable.**																
	Effect of parental divorce on the mediating variable					Effect of parental divorce and the mediating variable on cigarette dependence					Direct effect of parental divorce on cigarette dependence					Mediating effect of the mediator
	Beta	t	p	95% BCa CI		Beta	t	p	95% BCa CI		Beta	t	p	95% BCa CI		
Parental divorce	4.46	9.25	**< 0.001**	3.51	5.40	2.01	10.30	**< 0.001**	1.62	2.39	2.65	13.10	**< 0.001**	2.26	3.05	32.17%
Child physical abuse						0.14	15.13	**< 0.001**	0.13	0.16						
Parental divorce	1.89	6.46	**< 0.001**	1.32	2.46	2.29	11.65	**< 0.001**	1.91	2.68	2.66	13.13	**< 0.001**	2.26	3.05	15.87%
Child sexual abuse						0.19	11.93	**< 0.001**	0.16	0.22						
Parental divorce	7.55	9.08	**< 0.001**	5.92	9.19	1.90	10.07	**< 0.001**	1.53	2.27	2.70	13.21	**< 0.001**	2.30	3.10	41.77%
Child psychological abuse						0.11	19.42	**< 0.001**	0.09	0.12						
Parental divorce	0.08	0.11	0.914	−1.46	1.63	2.65	13.32	**< 0.001**	2.26	3.04	2.65	13.10	**< 0.001**	2.26	3.05	–
Child neglect						0.05	7.97	**< 0.001**	0.04	0.06						
Parental divorce	3.33	6.41	**< 0.001**	2.31	4.35	2.44	12.26	**< 0.001**	2.05	2.83	2.73	13.52	**< 0.001**	2.33	3.12	115.37%
Bullying victimization						0.08	9.14	**< 0.001**	0.07	0.10						
**Model 2: Waterpipe dependence score as the dependent variable.**																
	Effect of parental divorce on the mediating variable					Effect of parental divorce and the mediating variable on waterpipe dependence					Direct effect of parental divorce on waterpipe dependence					Mediating effect of the mediator
	Beta	t	p	95% BCa CI		Beta	t	p	95% BCa CI		Beta	t	p	95% BCa CI		
Parental divorce	4.46	9.25	**< 0.001**	3.51	5.40	407	6.37	**< 0.001**	2.81	5.32	5.40	8.45	**< 0.001**	4.14	6.65	32.74%
Child physical abuse						0.30	9.52	**< 0.001**	0.24	0.36						
Parental divorce	1.89	6.46	**< 0.001**	1.32	2.46	4.55	7.19	**< 0.001**	3.31	5.79	541	8.47	**< 0.001**	4.15	6.66	18.79%
Child sexual abuse						0.45	8.73	**< 0.001**	0.35	0.55						
Parental divorce	7.55	9.08	**< 0.001**	5.92	9.19	4.05	6.34	**< 0.001**	2.80	5.31	5.53	8.57	**< 0.001**	4.26	6.79	36.36%
Child psychological abuse						0.20	10.64	**< 0.001**	0.16	0.23						
Parental divorce	0.08	0.11	0.914	−1.46	1.63	5.40	8.69	**< 0.001**	4.18	6.62	5.42	8.48	**< 0.001**	4.16	6.67	–
Child neglect						0.19	9.64	**< 0.001**	0.15	0.22						
Parental divorce	3.33	6.41	**< 0.001**	2.31	4.35	4.65	7.27	**< 0.001**	3.39	5.90	5.39	8.40	**< 0.001**	4.13	6.65	16.02%
Bullying victimization						0.22	7.53	**< 0.001**	0.17	0.28						

## Discussion

It was always been assumed that in comparison to children whose parents were separated, adolescents living with both parents in the same household had better social, emotional, and behavioral functioning [41–43] with lower rates of smoking dependence [42, 44]. In fact, adolescence is a critical period with many transitional key events [16] and a greater tendency to draw into substance abuse, whenever there is lack of effective parenting [45]. However, as abovementioned, it is noteworthy that not all adolescents who experienced parental divorce have the same risk of developing smoking dependence (cigarette or waterpipe) [8]. Therefore, our study appears to be the first one to investigate the association between parental divorce and smoking dependence amongst Lebanese adolescents while taking into consideration the plausible mediating effect of abuse and bullying victimization, two frequently encountered, nationally important social challenges.

Consistent with previous studies [10, 42, 44, 46–52], our findings showed higher rates of cigarette and waterpipe dependence among adolescents whose parents were divorced compared to those living with both parents. This could be explained by the fact that divorced parents are busy solving their own issues, compromising their parental role in supervising their children’s behavior, meeting their emotional needs, sculpting their social skills and coping mechanisms, and most importantly providing guidance on the long-term side effects associated with risky behaviors [4, 44, 53, 54]. Also, evidence shows that lack in rigorous parental monitoring will presumably expose adolescents to outing with substance-using peers [4, 55], hence dropping easier into a potentially harmful habit given that at this age they may not be mature enough to grasp the long-term consequences of smoking [9, 53, 56]. Another possible explanation would be that adolescents will try to grab their parent’s attention and restore the weakened parent-child bond by adopting a new risky behavior [4]. Additionally, in Middle Eastern countries, smoking dependence is prevalent, socially accepted, and takes part of the culture [4, 9]. This habit is perceived as a pleasant social experience, and a mean of escape, relaxation, mood enhancement, and amusement [57, 58]. At last, neurobiological studies proved that the dopaminergic reward system in the nucleus accumbens is disrupted in children whose parents are divorced, subsequently experiencing high rates of smoking dependence [10, 59].

Many studies have attempted to identify factors associated with nicotine addiction in adolescents [4, 9]. Our mediation analysis showed that bullying victimization and all types of abuse (except neglect) partially mediated the association between parental divorce and nicotine dependence. Regarding abuse and regardless of its type, our bivariate analysis showed that it was significantly and positively associated with nicotine dependence. Authors usually report high rates of psychiatric disorders, nicotine dependence, and other health-damaging behaviors among maltreated children, especially in those experiencing parental divorce, lack of family cohesion, and insufficient parental supervision [60]. Our findings came in line with many population-based studies and meta-analyses that reported a higher risk of smoking among abused children, regardless of the abuse’s type [47, 61–73]. In fact, these adverse childhood events can impose an inescapable detrimental impact on children behavioral, social, and psychological development [47]. Exposed children may benefit of the demonstrable psychoactive actions of nicotine [4] in self-medicating their affective disturbances, regulating their mood, and reducing stress and anxiety [4, 47]. As well, smoking dependence in similar life circumstances can be explained by the belief that nicotine is a coping device adopted as a positive reinforce to relax, terminate a state of dysphoria, reduce tension, socialize or even please others [19, 33]. After conducting the multivariable analysis, only psychological and physical abuse remained associated to waterpipe dependence. This came in contrast to what was reported by Naghavi et al. on the major impact of sexual abuse on this specific behavior [74]. However, regarding cigarette smoking, after multivariable analysis, both the psychological and physical abuses remained associated to it. These findings came in contrast to prior hypothesis stating that both types of abuse should be present concomitantly to predict smoking [75], but similar to what was theoretically grounded in previous studies [76–79]. In fact, these adverse life-experiences are believed to be stressful, traumatic, and multidimensional, with the ability to disrupt the victim’s physiologic coping mechanisms, thus pushing him to adopt smoking as a mean to reduce stress [47].

Regarding bullying victimization, our bivariate analysis, showed that it was significantly and positively associated with nicotine dependence. In fact, divorce can generate a feeling of shame and stigmatization among the involved vulnerable children, that is associated with the lack of parental care, support, and companionship. Hence, adolescents whose parents are divorced might experience communication difficulties with peers and a low self-esteem, making them more likely to fall prey to bullying [14, 41, 80–82]. Subsequently, a possible explanation of how bullying victimization may partially mediate the association between parental divorce and smoking dependence would be its highly devastating consequences. Bullied children might suffer of adaptation difficulties [14], mental health problems [41], externalized behaviors, and smoking dependence [14, 83]. In such circumstances, adolescents will try to earn their peers’ respect and boost their self-confidence by resorting to smoking. This behavior is appraised as an “aesthetic enjoyable experience” that could improve social cohesion, ameliorate interindividual communication [84, 85], and create more friendship opportunities [56]. As well, secondary to the repeated harmful, aggressive, and negative bullying episodes, nicotine will be used by victims for relaxation given its stimulating pharmacological effect on the dopamine and serotonin systems [33]. Subsequently, to introduce healthier coping strategies, and better communication skills, public health workers and school staff members should adopt an individualized behavioral approach when supporting adolescents who experienced parental divorce [14].

### Clinical implications

The current study provides many valuable contributions to the literature. With the high reported rates of smoking dependence amongst offspring of divorced parents, and the mediating role of child abuse and bullying victimization, public health campaigns should be encouraged to support children during and after the divorce period so they could handle the marriage dissolution as harmoniously as possible. As well, selective intervention programs for high-risk individuals should be adopted at schools or public health centers by psychologists, physicians, and health-managers. Such interventions will undoubtedly decrease the likelihood of nicotine addiction by offering close monitoring and tailored approaches, providing a “substitute attachment figure”, and improving children’s resilience and coping strategies. Additionally, a faster adaptation to change also requires home based interventions such as the support of wider families and grandparents, the participation in mediation sessions, and the encouragement of parents-children conversations. Lastly, strategic nation-wide awareness campaigns should be planned to reduce the divorce-related stigma thus minimizing its social burden.

Regarding bullying victimization, general practitioners and pediatricians must routinely educate parents about bullying consequences and enquire about siblings and peers bullying. As well, it is vital to build awareness about this rising problem and provide school-based training to allow early identification, adequate evidence-based management, and timely referral. This could be possible by organizing whole-school approaches that aim to create structured programs with engagement of different disciplines, yearly celebrating the “International Day Against Violence and Bullying at School” launched by “The United Nations Educational, Scientific and Cultural Organization” (UNESCO) [86], and adopting cooperative learning in which teachers promote positive peer interaction for better student engagement and educational achievement.

Regarding abuse, given the faced challenges in recognizing children who are victims of it, care should be taken whenever a mental-health history is taken. Nonjudgmental and cognitively appropriate questions about the feelings of safety and the relationships within the family, should be asked individually to each parent and child. If needed, any person involved in the child’s life such as teachers, siblings, and other care-providers should be interviewed. When suspecting child abuse, child protection personnel should be communicated to take corrective measures. Moreover, an interdisciplinary collaboration between therapists, judicial authorities, child welfare agencies, education professionals, and mental-health providers, is warranted to improve laws that protect children, develop assessment tools to identify vulnerable subjects in need of support, and adopt adequate treatment strategies.

At last, regarding nicotine dependence, its relationship with the Lebanese culture suggests that changing the commonly perceived symbolic meaning of smoking as a pleasant social experience, a mean of relaxation, and mood enhancement, could help us in the fight against this habit. This could be possible by implementing cultural changes using national anti-smoking policies, advertising, and educational campaigns, bans on smoking commercials, and higher taxes on tobacco products.

### Limitations

While analyzing our findings, it is vital to keep in mind some limitations. First, given that we are using a self-reported questionnaire that might be influenced by respondent’s level of interest, the possibility of self-reporting bias cannot be ruled out. Second, a residual confounding bias is also possible given that not all sociodemographic characteristics associated with smoking were considered in this paper such as ethnicity, parental smoking, age of smoking initiation, abuse of other substances, and living with mother or father. Furthermore, included participants were only school students, thus our findings could not be generalized to adolescents who dropped out from school and might be facing more stressful events, thus being at a higher risk of nicotine addiction. At last, this study used a cross-sectional design, which hinders it from determining causality, and evaluating the temporality of the exposure, mediators, and outcomes. Subsequent cohort studies might be needed to establish this relationship.

## Conclusion

Our findings provide evidence that adolescents whose parents were divorced had higher rates of cigarette and waterpipe dependence compared to those who were living with both parents, with a mediating role of child physical, sexual, and psychological abuse, and bullying victimization. Consequently, this study results may serve as a first step towards enrolling separated parents and their children in special prevention programs to help them create a protective and supportive environment. Meanwhile, further studies should focus on improving these programs not only to boost adolescents’ confidence and moral, but also to build a better society in the long run.

## Data Availability

The authors do not have the right to share any data information as per their institutions’ policies.

## Abbreviations

UNDP: United Nations Development Programme
BMI: Body Mass Index
LWDS: Lebanon Waterpipe Dependence Scale-11
FTND: Fagerström Test for Nicotine Dependence
IBS: Illinois Bully scale
CASRS: Child Abuse Self-Report Scale
